# Genome-wide association to body mass index and waist circumference: the Framingham Heart Study 100K project

**DOI:** 10.1186/1471-2350-8-S1-S18

**Published:** 2007-09-19

**Authors:** Caroline S Fox, Nancy Heard-Costa, L Adrienne Cupples, Josée Dupuis, Ramachandran S Vasan, Larry D Atwood

**Affiliations:** 1 The National Heart Lung and Blood Institute's Framingham Heart Study, Framingham, MA, USA; 2 Boston University Schools of Medicine and Public Health, Boston, MA, USA

## Abstract

**Background:**

Obesity is related to multiple cardiovascular disease (CVD) risk factors as well as CVD and has a strong familial component. We tested for association between SNPs on the Affymetrix 100K SNP GeneChip and measures of adiposity in the Framingham Heart Study.

**Methods:**

A total of 1341 Framingham Heart Study participants in 310 families genotyped with the Affymetrix 100K SNP GeneChip had adiposity traits measured over 30 years of follow up. Body mass index (BMI), waist circumference (WC), weight change, height, and radiographic measures of adiposity (subcutaneous adipose tissue, visceral adipose tissue, waist circumference, sagittal height) were measured at multiple examination cycles. Multivariable-adjusted residuals, adjusting for age, age-squared, sex, smoking, and menopausal status, were evaluated in association with the genotype data using additive Generalized Estimating Equations (GEE) and Family Based Association Test (FBAT) models. We prioritized mean BMI over offspring examinations (1–7) and cohort examinations (10, 16, 18, 20, 22, 24, 26) and mean WC over offspring examinations (4–7) for presentation. We evaluated associations with 70,987 SNPs on autosomes with minor allele frequencies of at least 0.10, Hardy-Weinberg equilibrium p ≥ 0.001, and call rates of at least 80%.

**Results:**

The top SNPs to be associated with mean BMI and mean WC by GEE were rs110683 (p-value 1.22*10^-7^) and rs4471028 (p-values 1.96*10^-7^). Please see http://www.ncbi.nlm.nih.gov/projects/gap/cgi-bin/study.cgi?id=phs000007 for the complete set of results. We were able to validate SNPs in known genes that have been related to BMI or other adiposity traits, including the *ESR1 *Xba1 SNP, *PPARG*, and *ADIPOQ*.

**Conclusion:**

Adiposity traits are associated with SNPs on the Affymetrix 100K SNP GeneChip. Replication of these initial findings is necessary. These data will serve as a resource for replication as more genes become identified with BMI and WC.

## Introduction

Cardiovascular disease (CVD) is the leading cause of morbidity and mortality in the United States, affecting roughly twelve million people and accounting for nearly one million deaths per year [1]. Although improvements in cardiovascular risk factor profiles have contributed to reductions in CVD mortality, an increasing prevalence of obesity may have slowed this rate of decline [2]. Obesity increases the risk of all-cause mortality [3], vascular disease [4], and non-vascular causes of death including certain cancers [5]. Genetic and environmental factors have been linked to obesity [6]. We have previously reported linkage to body mass index (BMI) on chromosomes 6q23 and 11q24 [7,8], waist circumference (WC) on chromosome 6q23 [9], and weight change on chromosome 20q13 [10] in the Framingham Heart Study. Additionally, multiple quantitative trait loci and candidate genes have been mapped to adiposity-related traits, as recently reviewed [11].

As part of the Framingham Heart Study 100K Project, we sought to test the relation of multiple adiposity-related traits with the Affymetrix one hundred thousand single nucleotide polymorphisms (SNP) chip. A broad range of phenotypes were studied and include BMI, WC, height, and radiographic quantification of subcutaneous (SAT) and visceral (VAT) fat. In this manuscript we focus on mean BMI and mean WC. We tested the relation of these traits to 70,987 SNPs.

## Methods

Participants from the Framingham Heart Study Original Cohort and Offspring Cohort underwent genotyping with the Affymetrix 100K GeneChip; details about the selection process and genotyping are provided in the Overview [12]. Participants (n = 1345) were genotyped for the Affymetrix GeneChip Human Mapping 100K SNP set. For the current analysis, phenotype data were available in 1341 participants for mean BMI and 1079 participants for mean WC. For this manuscript, we focused on mean BMI over offspring examinations (1–7) and cohort examinations (10, 16, 18, 20, 22, 24, 26) and mean WC over offspring examinations (4–7). We evaluated associations with 70,987 SNPs on autosomes with minor allele frequencies ≥ 0.10, Hardy-Weinberg equilibrium (HWE) p-value ≥ 0.001, and call rates ≥80%.

### Phenotype assessment

Body weight and height were measured at all 7 Offspring examination cycles, from 1971 to 2001 and chronologically corresponding to 7 Original cohort examinations (10, 16, 18, 20, 22, 24, 26); WC was measured at the level of the umbilicus at 4 Offspring examinations (4, 5, 6, and 7). BMI was calculated by taking the weight (in kilograms) over the height (in meters-squared). Mean BMI across 7 offspring examinations (1–7) and 7 cohort examinations (10, 16, 18, 20, 22, 24, 26) was obtained by taking the average of all available measurements; mean WC across 4 examinations was obtained by taking the average of all available offspring measurements. Covariates were also averaged over the exams at which the adiposity measures were available.

Subcutaneous and visceral fat volumes (SAT and VAT, respectively) were measured on a subset of individuals who took part in the Framingham Offspring Multi-Detector Computed Tomography Study between 2002 and 2005. Briefly, subjects underwent eight-slice multi-detector computed tomography imaging of the chest and abdomen in a supine position as previously described (LightSpeed Ultra, General Electric, Milwaukee, WI) [13]. SAT and VAT volumes were assessed (Aquarius 3D Workstation, TeraRecon Inc., San Mateo, CA) via manual tracing of the abdominal muscular wall that separates the visceral from the subcutaneous compartment, with excellent inter-reader variability of 0.99 for VAT and SAT, as previously reported [13].

### Genotyping

Genotyping was performed using the 100K Affymetrix GeneChip. Please see the Overview [12] for details.

### Statistical methods

In total, a maximum of 1341 genotyped participants with phenotype information were available for analysis. Residuals were created from multiple linear regression models to adjust traits for covariates; these residuals were created separately in the Original Cohort and Offspring, and in women and men separately. The standardized residuals from these regression models were used to create ranked normalized deviates, which were in turn used for genetic analyses. Adiposity traits were age-adjusted (age and age-squared) and then multivariable adjusted; details of multivariable adjustment for each trait are presented in Table 1. Only multivariable-adjusted results are presented in this manuscript. All association analyses were performed using generalized estimating equations (GEE) and family-based association testing (FBAT); variance component methods were used for linkage; details are provided in the Overview [12]. To consider concordance of results among correlated adiposity traits (see the third table in this article), we selected SNPs with significant association (p < 0.01 in GEE or FBAT analyses) for at least 6 out of 8 following weight-related traits: BMI at Offspring exams 1–7 and chronologically corresponding Cohort exams 10, 16, 18, 20, 22, 24, 26 and mean BMI from these exams, and computed a geometric mean GEE p-values across all 8 traits for FBAT and GEE separately. We evaluated associations with 70,987 SNPs on autosomes with minor allele frequencies of at least 0.10, HWE p ≥ 0.001, and call rates of at least 80%. Linkage analysis was performed using variance components methods on a subset of 100K markers in linkage equilibrium and Marshfield short tandem repeats; please see the Overview [12] for more details, including power calculations.

**Table 1:** Phenotype master trait table: exam cycle, and numbers of participants in family plates with phenotype

			Exam cycle/s		
**Phenotype**	**Number of traits§**	**Sample Size**	**Offspring**	**Cohort**	**Adjustment**

**Body Mass Index; men and women combined and separate**	46	529–1341	1–7; mean 1–7; change from 1–7	10, 16, 18, 20, 22, 24, 26; mean 10, 16, 18, 20, 22, 24, 26	*
**Weight change**	11	468–1115	Change from 1–7		*
**Weight; men and women combined and separate**	43	498–1342	1–7	10, 16, 18, 20, 22, 24, 26	*
**Height; men and women combined and separate**	25	529–1341	1–7	10, 16, 18, 20, 22, 24, 26	*
**Waist circumference; men and women combined and separate**	24	479–1252	4–7; mean 4–7; change from 4–7	20, 22, 23; change from 20–23;	**
**Subcutaneous fat by computed tomography**	2	654	7	-	*
**Visceral fat by computed tomography**	2	653	7	-	*
**Waist circumference by computed tomography**	2	664	7	-	*
**Sagittal diameter by computed tomography**	2	665	7	-	*

§Refers to number of traits within each group actually analyzed

*All models included age, age-squared, sex, smoking, menopause

**Models additionally adjusted for body mass index

## Results

All traits (n = 157), including relevant examination cycles and multivariable-adjustments, are presented in Table 1. Table 2a presents the top 25 p-values obtained via GEE for mean BMI and mean WC. The top SNPs to be associated with mean BMI and mean WC by GEE were rs110683 (p-value 1.22*10^-7^) and rs4471028 (p-values 1.9*10^-7^); Table 2b presents the top SNPs for the FBAT procedure. Additional results can be found on the following website: http://www.ncbi.nlm.nih.gov/projects/gap/cgi-bin/study.cgi?id=phs000007. Table 2c presents all LOD scores of at least 2.0. For mean BMI, we observed a peak LOD score of 2.3 on chromosome 2p16, whereas for mean WC, we observed a peak LOD score of 2.3 on chromosome 2q14.

**Table 2:** (a) Top association results for mean BMI and mean WC based on GEE p-value; (b) Top 25 association results for mean BMI and mean WC based on FBAT p-value; (c) LOD scores of at least 2.0 with accompanying LOD score 1.5 support interval for mean BMI and mean WC

Trait	SNP	Chromosome	Physical Position (Mb)	GEE p-value	FBAT p-value	Gene
**2a. Top association results for mean BMI and mean WC based on GEE p-value**						

Mean BMI	rs1106683	7	130910780	1.2*10^-07^	3.2*10^-04^	
Mean WC	rs4471028	8	75457530	2.0*10^-07^	3.2*10^-04^	*GDAP1*
Mean WC	rs4469448	8	75457665	2.6*10^-07^	0.003	*GDAP1*
Mean WC	rs6996971	8	75455047	4.9*10^-07^	0.002	*GDAP1*
Mean WC	rs10504576	8	75429234	7.1*10^-07^	0.005	*GDAP1*
Mean WC	rs1875517	3	118790257	1.5*10^-06^	0.002	
Mean BMI	rs1106684	7	130910920	1.6*10^-06^	0.002	
Mean BMI	rs1333026	13	65018785	8.1*10^-06^	0.209	
Mean BMI	rs2296465	10	3299647	1.5*10^-05^	0.262	
Mean BMI	rs1374489	5	19658900	1.6*10^-05^	0.348	*CDH18*
Mean WC	rs1466113	17	68676913	2.7*10^-05^	0.004	*SSTR2*
Mean BMI	rs10486301	7	18183021	2.7*10^-05^	0.016	
Mean BMI	rs2221880	5	19420500	3.0*10^-05^	0.072	
Mean WC	rs1456873	4	62130929	3.1*10^-05^	0.005	
Mean BMI	rs10513097	5	11406101	4.4*10^-05^	0.022	*CTNND2*
Mean BMI	rs2361128	19	62431059	4.4*10^-05^	0.004	*ZNF264*
Mean BMI	rs2942329	5	19429660	4.5*10^-05^	0.089	
Mean WC	rs4129319	4	88752564	4.7*10^-05^	0.003	*SPARCL1*
Mean BMI	rs10509361	10	77223192	5.2*10^-05^	0.044	*C10orf11*
Mean BMI	rs2967001	5	19422000	6.0*10^-05^	0.074	
Mean BMI	rs464766	3	162570294	6.1*10^-05^	0.072	*ADMP*
Mean BMI	rs10487263	7	123603987	7.0*10^-05^	0.036	
Mean BMI	rs4922571	11	31643025	7.0*10^-05^	0.062	*ELP4*
Mean BMI	rs7013836	8	5666540	8.2*10^-05^	0.103	
Mean BMI	rs7202384	16	13658225	8.2*10^-05^	0.002	

**2b. Top 25 association results for mean BMI and mean WC based on FBAT p-value**						

**Trait**	**SNP**	**Chromosome**	**Physical Location (Mb)**	**GEE p-value**	**FBAT p-value**	**Gene**

Mean WC	rs10488165	7	132594899	0.011	2.6*10^-06^	*SEC8L1*
Mean WC	rs2206682	6	56001938	0.011	4.2*10^-06^	*COL21A1*
Mean WC	rs2223662	6	56001756	0.013	5.1*10^-06^	*COL21A1*
Mean WC	rs953536	9	111569442	0.007	8.2*10^-06^	*C9orf84*
Mean WC	rs10517461	4	37789743	2.3*10^-04^	2.9*10^-05^	*TBC1D1*
Mean WC	rs7941883	11	123262095	0.084	3.0*10^-05^	*OR8D4|OR4D5|OR6T1*
Mean BMI	rs10503776	8	25765786	0.009	3.8*10^-05^	*EBF2*
Mean BMI	rs711702	3	22956280	0.143	4.0*10^-05^	
Mean WC	rs4312989	6	55918441	0.075	4.2*10^-05^	
Mean WC	rs10519381	5	113700141	0.01	4.4*10^-05^	*KCNN2*
Mean WC	rs10483872	14	75239061	0.018	4.5*10^-05^	*KIAA0998*
Mean WC	rs315711	9	111628006	0.008	5.4*10^-05^	*C9orf84*
Mean WC	rs4715571	6	55917006	0.03	5.9*10^-05^	
Mean WC	rs667463	9	111647315	0.004	7.0*10^-05^	*C9orf84*
Mean BMI	rs7320523	13	67552774	0.193	7.4*10^-05^	
Mean WC	rs3752591	22	40664016	0.001	8.5*10^-05^	*C22orf18*
Mean WC	rs1496389	5	113753570	0.018	9.8*10^-05^	*KCNN2*
Mean WC	rs1619682	7	133453958	0.14	1.0*10^-04^	*SLC35B4*
Mean BMI	rs10492197	12	66871874	0.038	1.0*10^-04^	*IFNG|IL26|IL22*
Mean WC	rs10501467	11	79913437	0.037	1.1*10^-04^	
Mean WC	SNP_A-1731932	1	24035982	0.152	1.1*10^-04^	
Mean BMI	rs10512326	9	103934100	0.063	1.2*10^-04^	*SMC2L1*
Mean BMI	rs7533902	1	97791249	0.029	1.2*10^-04^	*DPYD*
Mean BMI	rs2870950	12	66870973	0.034	1.2*10^-04^	*IFNG|IL26|IL22*
Mean WC	rs2226351	21	25259810	1.8*10^-04^	1.3*10^-04^	

**2c. LOD scores of at least 2.0 with accompanying LOD score 1.5 support interval for mean BMI and mean WC**						

**Trait**	**SNP**	**Chromosome**	**Physical Position (bp)**	**LOD**	**LOD1.5 Lower Bound (bp)**	**LOD1.5 Upper Bound (bp)**

Mean BMI	rs9309153	2	48856798	2.3125	44048379	64579948
Mean WC	rs1992901	2	121386899	2.2721	116579748	132195205
Mean BMI	rs10518418	1	89149134	2.0053	51727132	99668198

Table 3 presents the top 25 SNPs for our multiple traits analysis, summarizing concordance of results in related BMI traits. The SNP with the lowest p-value was rs1106683 (p-value 3.8*10^-6^). We also evaluated 4 well-replicated genes in the obesity field (*ADIPOQ *[14], *ESR1 *[15], *LEP *[16], and *PPARG *[17]), as well as the recently identified *INSIG2 *gene [18]; Table 4a displays the associated validated SNPs from the literature that are either present in the Affymetrix 100K or that are in linkage disequilibrium (LD) with these SNPs. We found significant results for a SNP in LD with the *ESR1 *Xba1 SNP (rs3853250; FBAT p-value for mean BMI = 0.047). We also confirmed the association between a SNP in the *INSIG2 *gene (rs7566605; GEE p-value 0.001 for mean BMI) previously identified in this sample using a different analytic method [18]. We further explored associations with all SNPs in the Affymetrix 100K either within these genes or within 200 kb of these genes (Table 4b); only associations with p < 0.05 are presented. We identified 3 additional associated SNPs in the *INSIG2 *gene, 5 SNPs in the *PPARG *gene, 1 SNP in the *ADIPOQ *gene, and 5 SNPs in the *ESR1 *gene. Of the 4 SNPs present in the *LEP *gene, there were no associations with a p-value < 0.05.

**Table 3:** Results informed by combination of GEE and FBAT based on p-value of ≤0.01 for GEE or FBAT for 6 out of 8 BMI traits

SNP	Chromosome	Physical Position (bp)	Gene name	Mean GEE geometric p-value
rs1106683	7	130910780		3.8*10^-6^
rs2296465	10	3299647		5.9*10^-5^
rs10513097	5	11406101	*CTNND2*	6.8*10^-5^
rs2361128	19	62431059	*ZNF264|AURKC*	7.9*10^-5^
rs1374489	5	19658900	*CDH18*	1.1*10^-4^
rs10486301	7	18183021		1.3*10^-4^
rs1333026	13	65018785		1.3*10^-4^
rs10509361	10	77223192	*C10orf11*	1.7*10^-4^
rs10504368	8	64947097		2.7*10^-4^
rs947599	10	95256673	*C10orf3*	3.6*10^-4^
rs2012187	5	11375037	*CTNND2*	3.6*10^-4^
rs6480902	10	80157318		3.9*10^-4^
rs336583	3	162564683	*ADMP*	3.9*10^-4^
rs9290065	3	162259666	*PPMIL*	4.1*10^-4^
rs10494810	1	196868239	*NR5A2*	4.3*10^-4^
rs2012064	7	18220564		4.5*10^-4^
rs1869731	8	63972241	*FLJ39630*	4.7*10^-4^
rs775748	3	77679150	*ROBO2*	4.8*10^-4^
rs10499068	6	113189197		4.9*10^-4^
rs10236525	7	18184912		5.5*10^-4^
rs9309770	3	77647000	*ROBO2*	5.5*10^-4^
rs1504294	3	68831828	*FAM19A4*	5.5*10^-4^
rs7142517	14	54376554	*SAMD4|GCH1*	5.5*10^-4^
rs2051545	16	13680100		5.6*10^-4^
rs910623	1	115336446	TSPAN2	5.8*10^-4^

**Table 4:** (a) Comparison of mean BMI and mean WC Results with prior literature for SNPs that are either present in the 100K or in LD with a SNP in the 100K; (b) Associations of mean BMI and mean WC with all SNPs in or near genes (up to 200 kb away) for 5 well-replicated genes in the published literature (INSIG, PPARG, ADIPOQ, ESR1, LEP)* with a p-value < 0.05 in either FBAT or GEE**

4a. Comparison of mean BMI and mean WC Results with prior literature for SNPs that are either present in the 100K or in LD with a SNP in the 100K										
							**Mean BMI p-value**		**Mean WC p-value**	
							
**Gene**	**Candidate SNP**	**100K SNP**	**Location Candidate SNP (bp)**	**Location 100K SNP (bp)**	**D. prime**	**r^2^**	**FBAT**	**GEE**	**FBAT**	**GEE**

*INSIG2*	rs7566605	Rs7566605	118552255	118552255	1	1	0.449	**0.001**	0.975	0.480
*ESR1-Xba1*	rs9340799	rs3853250	152255495	152252014	1	0.62	**0.047**	0.350	0.980	0.105
	rs9340799	rs3853251	152255495	152252870	1	0.96	0.309	0.963	0.333	0.336
*LEP*	rs1349419	rs10487506	127471164	127472106	1	0.69	0.422	0.583	0.788	0.848
	rs12535747	rs10487505	127472286	127454114	0.83	0.34	0.188	0.286	0.420	0.975
	rs12535747	rs10487506	127472286	127472106	1	0.48	0.422	0.583	0.788	0.848
*PPARG*	rs1801282	rs1801282	12368125	12368125	1	1	0.556	0.178	0.290	0.406

4b. Associations of mean BMI and mean WC with all SNPs in or near genes (up to 200 kb away) for 5 well-replicated genes in the published literature (INSIG, PPARG, ADIPOQ, ESR1, LEP)* with a p-value < 0.05 in either FBAT or GEE**										

			**Mean BMI p-value**		**Mean WC p-value**					
							
**Gene**	**SNP**	**Physical Position (bp)**	**FBAT**	**GEE**	**FBAT**	**GEE**				

*INSIG*	rs9284719	118395025	0.148	**0.035**	0.510	0.925				
	rs3771942	118425080	0.766	**0.005**	0.812	0.984				
	rs10490628	118446520	0.464	**0.021**	0.765	0.238				
	rs7566605	118552255	0.449	**0.001**	0.975	0.480				
*PPARG*	rs2938392	12409608	0.158	**0.003**	0.644	0.244				
	rs709157	12437024	0.602	0.106	0.091	**0.023**				
	rs10510422	12505413	0.806	0.546	**0.044**	0.720				
	rs10510423	12526881	0.986	0.753	**0.038**	0.787				
	rs2454431	12558068	0.806	**0.034**	0.268	0.882				
	rs963163	12632067	0.997	**0.047**	0.749	**0.036**				
*ADIPOQ*	rs1042464	187878274	0.962	0.231	**0.024**	0.722				
*ESR1*	rs851982	152117099	0.880	0.538	0.119	**0.043**				
	rs10484922	152224431	0.318	**0.012**	0.067	0.528				
	rs3853250	152252014	**0.047**	0.350	0.980	0.105				
	rs3778099	152510689	**0.033**	0.367	0.513	0.689				
	rs9322361	152551257	**0.020**	0.096	0.122	0.522				

*No SNPs in the *LEP *gene had a p-value < 0.05

**The following number of SNPs were evaluated in the *INSIG*, *PPARG*, *ADIPOQ*, *ESR1*, and *LEP *genes: 19, 28, 16, 37, 4

### Additional Findings

We also identified several additional SNPs in genes in relation to mean BMI or mean WC among our list of the top 500 SNPs http://www.ncbi.nlm.nih.gov/projects/gap/cgi-bin/study.cgi?id=phs000007. The *LRP1B *gene (SNP rs3923350, GEE p-value 0.0005) was associated with both mean BMI and mean WC. We also found association with the *VIP *gene (SNP rs620598, GEE p = 0.001), the *LEPR *gene (SNP rs2025804, GEE p-value = 0.003), the *ADRB1 *gene (SNP rs6585258, FBAT p-value 0.004), the *NPY2R *gene (SNP rs2880411, p-value = 0.006), the *HSD3B1 *gene (rs 4659200, FBAT p-value 0.0007), the *ADRA1B *gene (SNP rs952037, GEE p-value = 0.002), *IL6R *(SNP rs4129267, FBAT p-value = 0.003), *AGTR1 *(SNP rs275678, FBAT p-value = 0.006), and *FSHR *(SNP rs1504155; GEE p-value = 0.0004).

## Discussion

In our analysis of adiposity-related traits, we found strong and significant results to SNPs on the Affymetrix 100K GeneChip. Further, we have confirmed or replicated several well-validated genes that have been reported to be related to adiposity.

One of the top SNPs that we identified via the GEE method is located in the *SSTR2 *gene, the somatostatin receptor 2 gene, which has been reported to suppress growth hormone secretion. We also identified several additional SNPs in genes in relation to mean BMI or mean WC among our list of the top 500 SNPs. The *LRP1B *gene is a member of the LDL receptor gene family, and represents a potentially attractive candidate gene. The *VIP *gene (SNP rs620598, GEE p = 0.001) is a member of the glucagon family that plays a role in multiple physiologic and metabolic pathways, including myocardial contractility, smooth muscle relaxation, blood pressure lowering and vasodilation, and glycogenolysis. We also found significant associations with multiple genes that have been previously associated with adiposity-related traits [11].

Using our clustered traits analysis, we identified the *CTNND2 *gene, a gene that is part of the catenin family that may be involved in nutrient absorption in the intestine and signaling with nuclear receptors including PPAR [19]. We also identified the *NR5A2 *gene, a gene that is part of the nuclear receptor subfamily, a family of orphan receptors. *NR5A2 *is a key regulator of CYP7A expression in the liver, and *PPM1L *(protein phosphatase 1), a gene that is a suppressor of the SAPK pathway and may be involved in oxidative stress and apotosis.

### Well-replicated candidate genes

We were able to confirm association (i.e. validate) with the *INSIG2 *gene (SNP rs7566605, GEE p-value = 0.001). This same SNP was previously identified in association with BMI in this same sample using a different analytic method [18]. We also had nominal significance with a SNP in LD with the *ESR1 *Xba1 SNP, and multiple other SNPs in well-replicated obesity genes, suggesting that the Affymetrix 100K GeneChip provides a valid tool for uncovering candidate gene associations with adiposity-related traits. Of note, some of our SNPs did overlap with results reported for BMI using different analytic methods (Herbert et al, http://gmed.bu.edu/about/index.html[20]).

### Comparison with prior linkage results

We have previously identified a locus for BMI on chromosome 1 (D1S1665, LOD score 1.85) [7]. This peak falls within the 1.5 LOD score interval for our current finding on chromosome 1. We also have previously identified a LOD score of 2.0 for waist circumference on chromosome 2q14 [9], nearby to our current LOD score of 2.27 for mean WC. Differences with previously reported results may stem from our use of different phenotypes.

### Strengths and limitations

Strengths of our study lie in our assessment of multiple measures of BMI and WC in a sample unselected for these traits, thus improving precision. We also have excellent assessment of potential confounders that we are able to adjust for in our residual creation. Because the Framingham Heart Study has measured multiple traits, we are able to examine trait clustering, which may be more likely to identify SNPs in coding regions. Limitations exist as well. Our sample is neither ethnically diverse nor nationally representative, and it is uncertain how our results would apply to other ethnic groups. However, in genetics studies, sample homogeneity is beneficial in order to reduce population stratification. Further, none of these results reached genome-wide significance; please see the Overview [12] for details regarding this threshold. These results should be considered preliminary, and are likely to contain false negatives and false positives. Therefore, replication in independent samples is critical. For limitations pertaining to our genotyping or statistical methods, including multiple testing, please see the Overview [12].

## Conclusion

Adiposity-related traits are associated with SNPs on the Affymetrix 100K SNP GeneChip. Further work to replicate some of these SNPs in other samples is necessary. These data will serve as a resource for replication as more genes become identified with BMI and WC.

## Abbreviations

BMI = body mass index; CVD = cardiovascular disease; FBAT = Family Based Association Test; GEE = Generalized Estimating Equations; HWE = Hardy-Weinberg equilibrium; LD = linkage disequilibrium; SAT = subcutaneous fat; SNP = single nucleotide polymorphisms; VAT = visceral fat; WC = waist circumference.

## Competing interests

The authors declare that they have no competing interests.

## Authors' contributions

CF drafted the manuscript and interpreted the data. JD assisted in the design of the study and in performing the statistical analyses. LAC contributed to the analytical design, the interpretation of these results and to edits of the manuscript. NHC contributed to the analytical design and the phenotype creation. RV contributed to the phenotype acquisition, the interpretation of the results, and edits to the manuscript. LA contributed to the design, analysis, and interpretation of the findings. All authors gave approval to the final version of the manuscript.
